# Advanced Glycation End Products Induce Human Corneal Epithelial Cells Apoptosis through Generation of Reactive Oxygen Species and Activation of JNK and p38 MAPK Pathways

**DOI:** 10.1371/journal.pone.0066781

**Published:** 2013-06-12

**Authors:** Long Shi, Xiaoming Yu, Hongling Yang, Xinyi Wu

**Affiliations:** 1 Department of Ophthalmology, Qilu Hospital of Shandong University, Jinan, China; 2 The Key Laboratory of Cardiovascular Remodeling and Function Research, Chinese Ministry of Education and Chinese Ministry of Health, Qilu Hospital of Shandong University, Jinan, China; Institut national de la santé et de la recherche médicale - Institut Cochin, France

## Abstract

Advanced Glycation End Products (AGEs) has been implicated in the progression of diabetic keratopathy. However, details regarding their function are not well understood. In the present study, we investigated the effects of intracellular reactive oxygen species (ROS) and JNK, p38 MAPK on AGE-modified bovine serum albumin (BSA) induced Human telomerase-immortalized corneal epithelial cells (HUCLs) apoptosis. We found that AGE-BSA induced HUCLs apoptosis and increased Bax protein expression, decreased Bcl-2 protein expression. AGE-BSA also induced the expression of receptor for advanced glycation end product (RAGE). AGE-BSA-RAGE interaction induced intracellular ROS generation through activated NADPH oxidase and increased the phosphorylation of p47phox. AGE-BSA induced HUCLs apoptosis was inhibited by pretreatment with NADPH oxidase inhibitors, ROS quencher N-acetylcysteine (NAC) or neutralizing anti-RAGE antibodies. We also found that AGE-BSA induced JNK and p38 MAPK phosphorylation. JNK and p38 MAPK inhibitor effectively blocked AGE-BSA-induced HUCLs apoptosis. In addition, NAC completely blocked phosphorylation of JNK and p38 MAPK induced by AGE-BSA. Our results indicate that AGE-BSA induced HUCLs apoptosis through generation of intracellular ROS and activation of JNK and p38 MAPK pathways.

## Introduction

Diabetes has become a public health problem of considerable magnitude [1].

Diabetic keratopathy has been recognized as a serious complication of diabetes [2], such as persistent corneal epithelial defects, recurrent corneal erosion, persistent corneal edema and delayed corneal epithelial wound repair. Particularly for diabetic retinopathy patients undergoing vitrectomy, the removal of the corneal epithelium during the procedure results in a considerable delay in corneal epithelial wound healing [3]. Proper healing of corneal epithelial wounds is vital for maintaining a clear cornea and preserving vision. Delayed healing of corneal epithelial wound may cause sight-threatening complications, such as ocular surface irregularity, microbial keratitis or even blindness. So far, there is no effective strategy for the treatment of diabetic keratopathy in clinical practice [4]. The mechanism of the disease is not completely understood. Therefore delineating the underlying mechanisms of diabetic keratopathy will be of great clinical value.

Advanced Glycation End Products (AGEs) has been found to play an important role in the development of diabetic complications, such as diabetic nephropathy, retinopathy and atherosclerosis [5], [6]. AGEs are a heterogeneous group of irreversible adducts from glucose-protein condensation reactions, as well as lipids and nucleic acids exposed to reducing sugars [7]. Initially, there is formation of reversible Schiff base intermediates (Amadori’s product), which undergoes a complex series of chemical rearrangements, to yield irreversible AGEs [8]. The formation and accumulation of AGEs have been demonstrated to progress at an accelerated rate under diabetic conditions [9]. It is widely accepted that AGEs play an important role in diabetic keratopathy [10], [11]. The accumulation of AGEs has been detected at the site of the corneal epithelium and epithelial basement membrane in diabetic rats [12], [13], monkeys [14] and patients [10]. It has been shown that AGEs was elevated in tears of diabetic patients [15]. Moreover, treatment with aminoguanidine, an AGEs inhibitor, prevented corneal structural abnormalities in diabetic rats [11], [16]. Although these observations suggest that AGEs accumulation has an important role in the progression of diabetic keratopathy. However, details regarding their function are not well understood.

The biological properties of AGEs have been associated with their ability to interact with the receptor for AGEs (RAGE) [17]. RAGE is a signal transduction receptor of the immunoglobulin superfamily [18]. AGEs-induced tubular epithelial-to-mesenchymal transition (EMT) and renal fibrosis were RAGE dependent [19]. AGE-RAGE axis appears to play a central role in the inflammation, neurodegeneration, and retinal microvascular dysfunction occurring during diabetic retinopathy [20]. Previous study has found that RAGE expression was greater in corneal epithelial cells of diabetic rats than in those of control rats [21].

Apoptosis is a potential mechanism through which AGEs exert effects. It has been shown that AGEs induced apoptosis in renal mesangial cells, vascular endothelial cells and retinal pericytes [22], [23], [24]. Apoptosis in corneal epithelium has been demonstrated in diabetic rat [12], [13], [25], in which the accumulation of AGEs is implicated. Increases in corneal epithelial cells apoptosis contributes to delayed epithelial wound healing in diabetic cornea. The generation of intracellular reactive oxygen species (ROS) has been shown to mediate cellular responses to AGEs [26]. ROS such as superoxide anion, hydroxyl radicals and hydrogen peroxide, can initiate inappropriate or altered cellular signal transduction pathways and cause toxicity [27]. Excessive production of ROS plays a important role in apoptosis [28]. It has been reported that AGEs induced retinal pericyte apoptosis through overproduction of intracellular ROS [24]. AGEs have been reported to activates Mitogen-activated protein kinase (MAPK) pathways [29]. MAPK pathways are a family of serine-threonine protein kinases [30]. C-jun N terminal kinase (JNK) and p38 MAPK constitute two major subfamilies of MAPK pathways that can participate in apoptosis [31]. There is evidence that AGEs induced osteoblast apoptosis via JNK and p38 MAPK [32].

Based on these findings, it was hypothesized that AGEs-RAGE interaction induce intracellular ROS generation and activate JNK and p38 MAPK, which contribute to corneal epithelium apoptosis. In the present study, we investigated whether AGE-modified bovine serum albumin (BSA) could induce apoptosis in Human telomerase-immortalized corneal epithelial cells (HUCLs), and determined the effect of intracellular ROS and JNK, p38 MAPK on AGE-BSA induced HUCLs apoptosis.

## Materials and Methods

### Reagents

Bovine serum albumin (BSA) was obtained from Sigma-Aldrich (St Louis, MO). Antibodies against Bax and Bcl-2 were purchased from Santa Cruz Biotechnology, Inc. (Santa Cruz, CA). Antibodies against JNK, p38 MAPK, phospho-JNK (Thr183/Tyr185) and phospho-p38 MAPK (Thr180/Tyr182) were obtained from Cell Signaling Technology, Inc. (Danvers, MA). Antibodies against RAGE were obtained from R&D Systems (Abingdon, U.K.). SP600125 was obtained from A. G. Scientific, Inc (San Diego, CA), SB203580 was purchased from Calbiochem (San Diego, CA).

### Preparation of AGE proteins

AGE-BSA was prepared as previously described with minor modifications [33]. Briefly, 50 mg/ml BSA was incubated under sterile conditions with 0.5 mol/l D-glucose in 0.1 mol/l phosphate buffered saline (PBS, pH 7.4) at 37°C for 10 weeks in the dark. After incubation, preparations were extensively dialyzed against PBS to remove free glucose. Unmodified BSA was incubated under the same conditions without glucose as a control. Protein concentrations were determined by the Bradford method. Endotoxin concentrations were measured by the limulus amebocyte lysate assay (EToxate; Sigma-Aldrich, St Louis, MO), and no endotoxin was detected.

### Estimation of AGE formation

AGE-BSA was characterized based on lysine residue modifications and their fluorescence properties. The fraction of modified lysine residues was measured by means of the 2,4,6-trinitrobenzenesulfonic acid method (TNBS, Sigma-Aldrich, St. Louis, Mo) that estimates the proportion of unmodified lysine in AGE-BSA preparation compared with that of the unmodified BSA. By this method, we showed that the extent of lysine modification in our preparation of AGE-BSA was 84% compared to unmodified BSA. Extent of fluorescent AGEs formation was assessed spectrofluorometrically. AGE-BSA and unmodified BSA was diluted with PBS, and fluorescence intensity was recorded at excitation 360 nm, emission 450 nm. The characteristic glycation fluorescence of AGE-BSA was increased approximately 12-fold compared with unmodified BSA. This indeed strongly suggested that AGEs have been formed.

N^ε^-carboxy-methyl-lysine (CML) was the major forms of AGEs in vivo. CML concentration was measured by an ELISA (Uscn Life Science Inc., Wuhan, China ). After glycation, AGE-BSA was characterized by a 59-fold higher CML concentration compared to the unmodified BSA (11.8 nmol/mg protein in AGE-BSA versus 0.2 nmol/mg protein in unmodified BSA). We therefore examined the effects of 50, 100, and 200 µg/ml AGE-BSA on HUCLs, since these CML concentrations are representative of those found in the plasma of diabetic patients [34], [35].

### Cell culture

HUCLs were kindly provided by Professor Fu-Shin X. Yu (School of Medicine, Wayne State University, USA) [36]. HUCLs were cultured in defined keratinocyte serum free medium (Invitrogen, CA, USA) in a humidified 5% CO2 incubator at 37°C. Cells were seeded into 6-well plates at a density of 2×10^5^ cells per well in normal growth medium.

### Detection of apoptosis

Apoptosis was investigated with the Annexin V- fluorescein isothiocyanate (FITC) Apoptosis Detection Kit (BioVision Inc., Mountain View, CA, USA), following the manufacturer’s instructions.

### Western blot analysis

Western blotting proceeded as previously described [37]. Briefly, cultured cells were collected at indicated time and lysed by shaking at 4°C for 30 min in RIPA buffer (50 mM Tris-HCl, 0.25% Na-deoxycholate, 1% NP-40, and 150 mM NaCl, NaF and 1 mM Na_3_VO_4_) containing protease inhibitors. Cell lysates were centrifuged at 12,000 g for 15min at 4°C. The supernatant was boiled for 5min. Total protein was quantified and protein samples were subjected to 10% sodium dodecyl sulfate-polyacrylamide gel electrophoresis, and then transferred to nitrocellulose membranes. The membranes were blocked with 5% skim milk in Tris-buffered saline for 2 h at room temperature before overnight incubation at 4°C with primary antibodies. Nitrocellulose membranes were extensively washed with Tris-buffered saline and incubated with secondary antibodies for 2 h at 37°C. Protein bands were visualized using enhanced chemiluminescence as described by the supplier. Densitometric analysis has been carried out with Quantity One software (Bio-Rad, CA, USA).

### Measurement of intracellular ROS

Intracellular ROS levels were determined by measuring the DCFH-DA as previously described [38]. Briefly, cells were incubated for 30 min with 10 µM DCFH-DA (Sigma-Aldrich, St Louis, MO, USA) at 37°C in the dark, and then treated as indicated. Intracellular ROS levels were analyzed by using a fluorometer with 485 nm excitation and 535 nm emission wavelengths. Nonglycated BSA was used as a control. The data are means from experiments performed in triplicate. The intracellular accumulation of ROS was also imaged on an laser scanning confocal system on an inverted fluorescence microscope.

### Determination of NADPH oxidase activity

Nicotinamide adenine dinucleotide phosphate (NADPH) oxidase activity was determined as previously described [39]. Briefly, HUCLs were treated as indicated and then were suspended in homogenization buffer (20 mM Hepes, pH 7.0, 1 mM EDTA, and 100 mM KCl containing protease inhibitor mixtures). NADPH oxidase activity was measured by luminescence assay in 50 mM PBS, pH 7.0, containing 150 mM sucrose, 1 mM EGTA, 5 mM lucigenin as the electron acceptor, and 100 mM NADPH as the substrate. Assays were carried out in the dark at room temperature.

### Statistical analysis

Results were expressed as means ±SEM. Pairwise comparisons were evaluated by the Student–Newman–Keuls procedure or Dunnett’s T3 procedure when the assumption of equal variances did not hold. *P*<0.05 was considered statistically significant. Data analysis was carried out with the Statistical Package for Social Sciences (SPSS version 11.0).

## Results

### AGE-BSA induced apoptosis of cultured HUCLs

**Figure 1:**
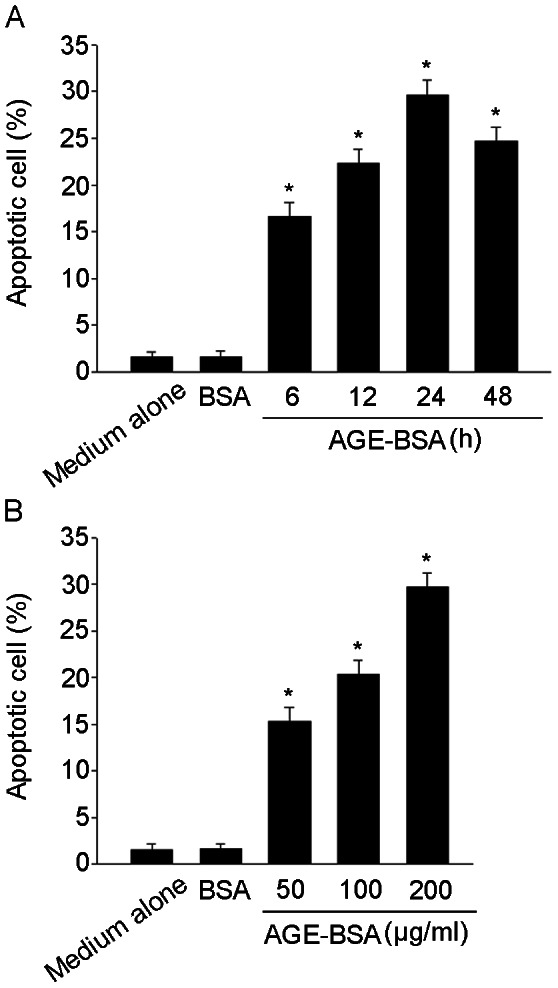
AGE-BSA induced apoptosis of cultured HUCLs. Apoptosis was determined by flow cytometer. (A) HUCLs were incubated with AGE-BSA (200 µg/ml) for 6, 12, 24 and 48 h. (B) HUCLs were treated with 50, 100 and 200 µg/ml of AGE-BSA for 24 h. Data are representative of three independent experiments. *, *P*<0.05 vs. medium alone group.

We investigated whether AGE-BSA could induce apoptosis in HUCLs. HUCLs were incubated with 200 µg/ml of AGE-BSA for 6, 12, 24 and 48 h or treated with 50, 100 and 200 µg/ml of AGE-BSA for 24 h. Apoptosis was determined by flow cytometer. As shown in Figure 1, exposure of HUCLs to AGE-BSA induced a time- and dose-dependent increase in apoptosis.

### AGE-BSA induced Bax protein expression and inhibited Bcl-2 protein expression in HUCLs

**Figure 2:**
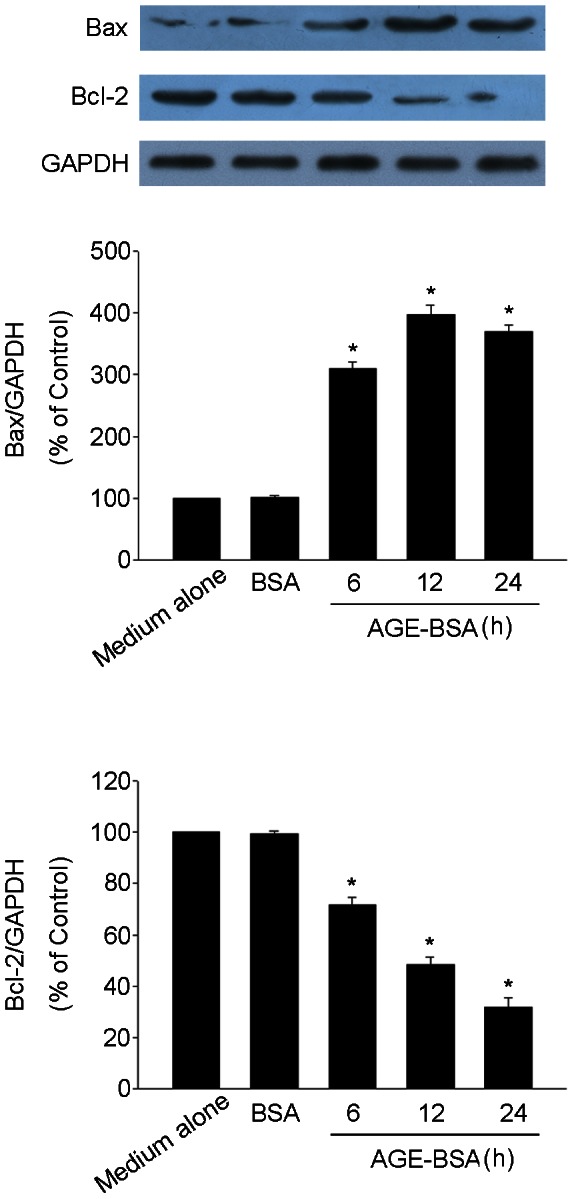
AGE-BSA induced Bax protein expression and inhibited Bcl-2 protein expression in HUCLs. HUCLs were incubated with AGE-BSA (200 µg/ml) for 6, 12 and 24 h, Western blot analysis of Bax and Bcl-2 levels in the whole cell extracts. Data are representative of three independent experiments. *, *P*<0.05 vs. medium alone group.

To examine the potential mediators of AGE-BSA-induced apoptosis, pro-apoptotic molecule Bax and anti-apoptotic molecule Bcl-2 expression were analyzed by western blot. As shown in Figure 2, AGE-BSA significantly increased Bax protein expression. In contrast, AGE-BSA markedly inhibited Bcl-2 protein expression. These results suggest that AGE-BSA-induced HUCLs apoptosis was associated with increase Bax expression and decrease Bcl-2 expression.

### AGE-BSA induced RAGE expression in HUCLs

**Figure 3:**
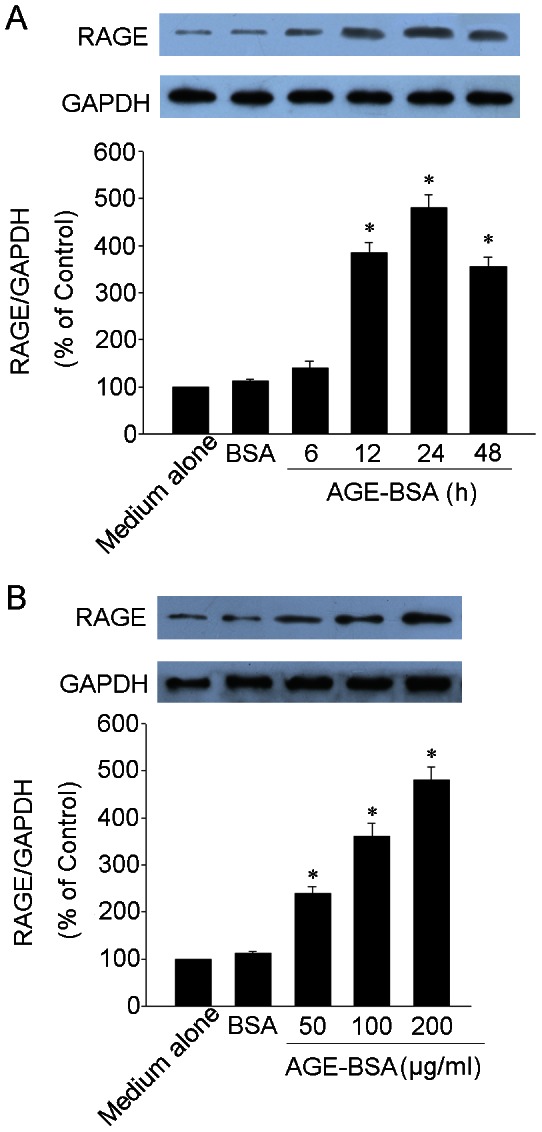
AGE-BSA induced RAGE expression in HUCLs. Expression of RAGE protein was determined by Western blot analysis. (A) HUCLs were incubated with AGE-BSA (200 µg/ml) for 6, 12, 24 and 48 h. (B) HUCLs were treated with 50, 100 and 200 µg/ml of AGE-BSA for 24 h. Data are representative of three independent experiments. *, *P*<0.05 vs. medium alone group.

We next investigated the effect of AGE-BSA on RAGE expression in HUCLs. HUCLs were incubated with 200 µg/ml of AGE-BSA for 6, 12, 24 and 48 h or treated with 50, 100 and 200 µg/ml of AGE-BSA for 24 h. Expression of RAGE protein was determined by Western blot analysis. As shown in Figure 3, exposure of HUCLs to AGE-BSA induced a time- and dose-dependent increase in expression of RAGE protein.

### AGE-BSA increased NADPH-dependent intracellular ROS generation in HUCLs

**Figure 4:**
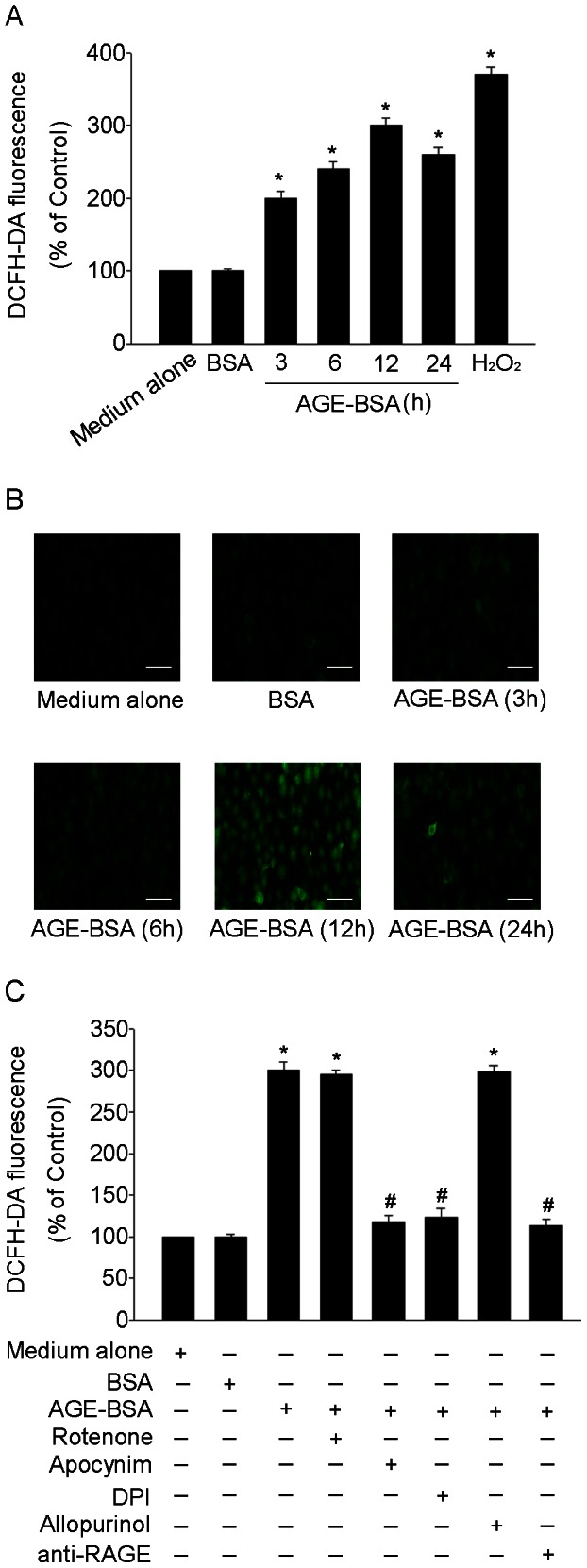
AGE-BSA increased NADPH-dependent intracellular ROS production in HUCLs. Intracellular ROS production was assessed using a DCFH-DA fluorescence. (A) HUCLs were treated with AGE-BSA (200 µg/ml) for 3, 6, 12 and 24 h, Hydrogen peroxide (1 mM) was added to cells as a positive control. (B) Intracellular ROS generation was visualized under a laser scanning confocal microscope. Scale bar  = 50 µm. (C) HUCLs were pretreated with DPI (NADPH oxidase inhibitor, 10 µM), apocynin (NADPH oxidase inhibitor, 300 µM), allopurinol (xanthine oxidase inhibitor, 10 µM), rotenone (inhibitor of mitochondrial electron transport complex I, 5 µM) or neutralizing anti-RAGE antibodies (20 µg/ml) for 1 h. and then incubated with AGE-BSA (200 µg/ml) for 12 h. Data are representative of three independent experiments. *, *P*<0.05 vs. medium alone group; #, *P*<0.05 vs. AGE-BSA group.

AGE-BSA exerts pro-apoptotic effect by inducing intracellular ROS production in a variety of cell types [24]. To elucidate the mechanism of AGE-BSA induced HUCLs apoptosis, the effects of AGE-BSA on intracellular ROS production were assessed. The level of intracellular ROS was detected based on DCFH-DA fluorescence. As shown in Figure 4 A, AGE-BSA induced an acute increase of intracellular ROS generation, and the earliest significant increase in intracellular ROS production was after 3h AGE-BSA treatment in HUCLs. AGE-BSA induced intracellular ROS production in HUCLs was confirmed by laser scanning confocal microscopy. As shown in Figure 4 B, AGE-BSA greatly increased the number of cells with high intensity of fluorescence compared with cells cultured in medium alone. Furthermore, AGE-BSA induced intracellular ROS production was almost completely blocked by pretreating HUCLs with inhibitors of NADPH oxidase, Diphenylene iodonium (DPI) or apocynin, but not by inhibitors of other potential enzymes, suggesting that AGE-BSA induced intracellular ROS production in HUCLs was dependent on NADPH oxidase (Figure 4 C). Next, we investigated whether AGE-BSA signal through RAGE to induce ROS generation. We used a neutralizing anti-RAGE antibody to block the AGE-RAGE interaction. As shown in Figure 4 C, anti-RAGE antibodies completely block the enhanced ROS generation by AGE-BSA, demonstrating that AGE-BSA bind RAGE to induce ROS generation.

### AGE-BSA activated NADPH oxidase in HUCLs

**Figure 5:**
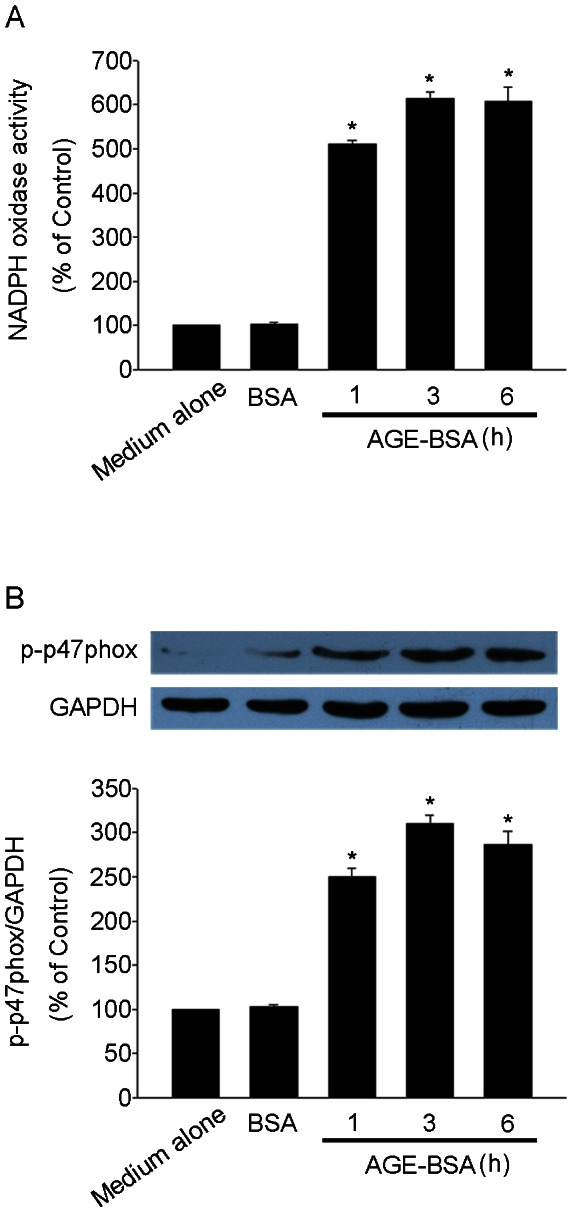
AGE-BSA activated NADPH oxidase in HUCLs. HUCLs were treated with AGE-BSA (200 µg/ml) for 1, 3 and 6 h. (A) NADPH oxidase activity was assessed. (B) Western blot analysis was used to detect p-p47phox expression. Data are representative of three independent experiments. *, *P*<0.05 vs. medium alone group.

To further investigate the mechanisms underlying the induction of intracellular ROS production by AGE-BSA, we examined the effect of AGE-BSA on the activity of NADPH oxidase. As shown in Figure 5 A, AGE-BSA led to a time-dependent increase of NADPH oxidase activity in HUCLs. p47phox is a major subunit of NADPH oxidase, it becomes phosphorylated and stimulates enzymatic activity. As shown in Figure 5 B, the levels of p-p47phox were significantly increased in HUCLs 1 h after treatment with AGE-BSA.

### Intracellular ROS generation mediated AGE-BSA-induced apoptosis in HUCLs

**Figure 6:**
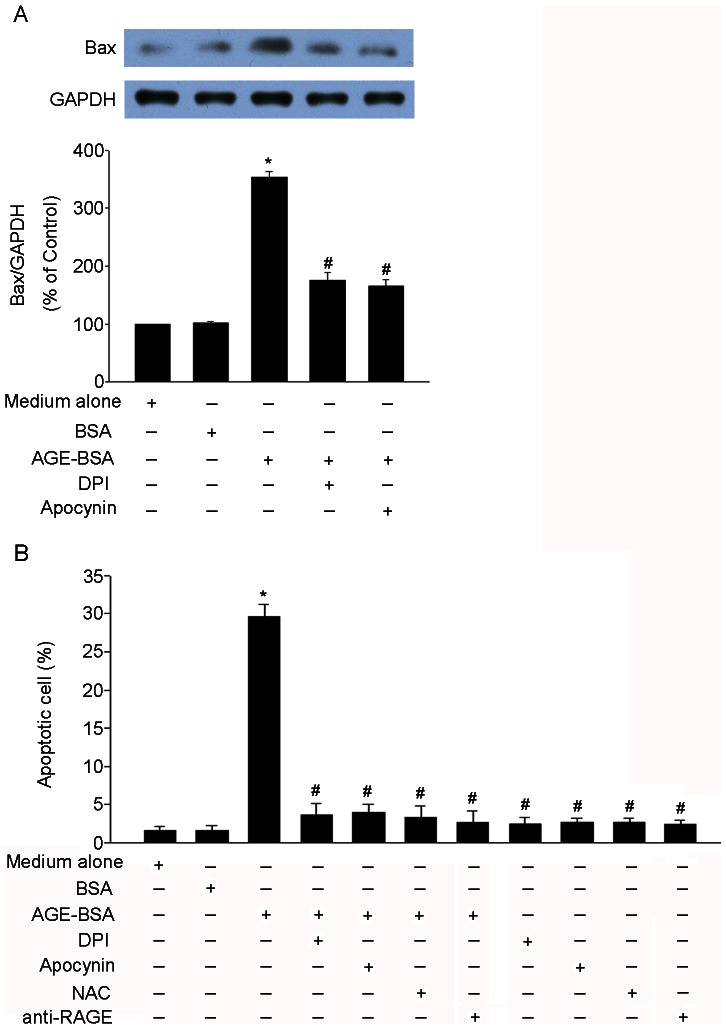
Intracellular ROS generation was required for AGE-BSA-induced apoptosis in HUCLs. (A) HUCLs were pretreated with DPI (10 µM) or apocynin (300 µM) for 1 h, and Bax protein expression was analyzed 24 h after AGE-BSA (200 µg/ml) treatment by Western blot. (B) HUCLs were pretreated with DPI (10 µM), apocynin (300 µM), NAC (20 µM) or neutralizing anti-RAGE antibodies (20 µg/ml) for 1 h, and apoptosis was analyzed 24 h after AGE-BSA (200 µg/ml) treatment by flow cytometer. Data are representative of three independent experiments. *, *P*<0.05 vs. medium alone group; #, *P*<0.05 vs. AGE-BSA group.

We then examined whether intracellular ROS generation was necessary for AGE-BSA-induced apoptosis in HUCLs. As shown in Figure 6, pretreatment of HUCLs with NADPH oxidase inhibitors (DPI or apocynin) significantly suppressed pro-apoptotic molecule Bax protein expression and apoptosis induced by AGE-BSA. Pretreatment of HUCLs with ROS scavenger N-acetylcysteine (NAC) significantly inhibited apoptosis induced by AGE-BSA. These results suggest that intracellular ROS generation through NADPH oxidase was required for AGE-BSA-induced apoptosis in HUCLs. In addition, AGE-BSA induced HUCLs apoptosis could be blocked by anti-RAGE antibodies, suggesting that AGE-BSA-induced apoptosis is mainly mediated by RAGE.

### AGE-BSA induced JNK and p38 MAPK phosphorylation in HUCLs

**Figure 7:**
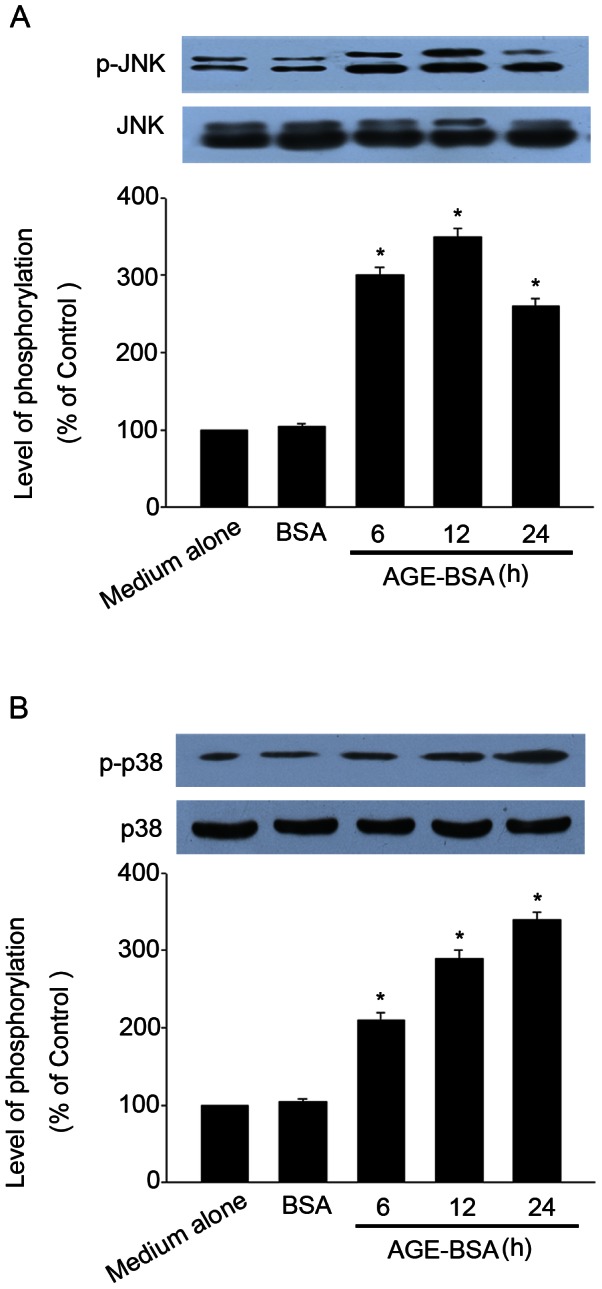
AGE-BSA induced JNK and p38 MAPK phosphorylation in HUCLs. HUCLs were incubated with AGE-BSA (200 µg/ml) for 6, 12 and 24 h. The total and phosphorylation of JNK (A) and p38 MAPK (B) were analyzed by Western blot. Data are representative of three independent experiments. *, *P*<0.05 vs. medium alone group.

Oxidant stress is known to activate MAPKs family, specifically JNK and p38 MAPK, by phosphorylation [40]. Since AGE-BSA induced intracellular ROS generation, we speculated that JNK and p38 MAPK could be involved in AGE-BSA-induced apoptosis. Thus, we investigated whether AGE-BSA could induce JNK and p38 MAPK phosphorylation in HUCLs. HUCLs were treated with 200 µg/ml of AGE-BSA for 6, 12 and 24 h, followed by extraction of the cellular protein. The expressions of total and phosphorylated JNK and p38 MAPK were determined by Western blot analysis. As shown in Figure 7, HUCLs stimulated with AGE-BSA induced increase in the phosphorylation of JNK and p38 MAPK.

### JNK and p38 MAPK mediated AGE-BSA-induced apoptosis in HUCLs

**Figure 8:**
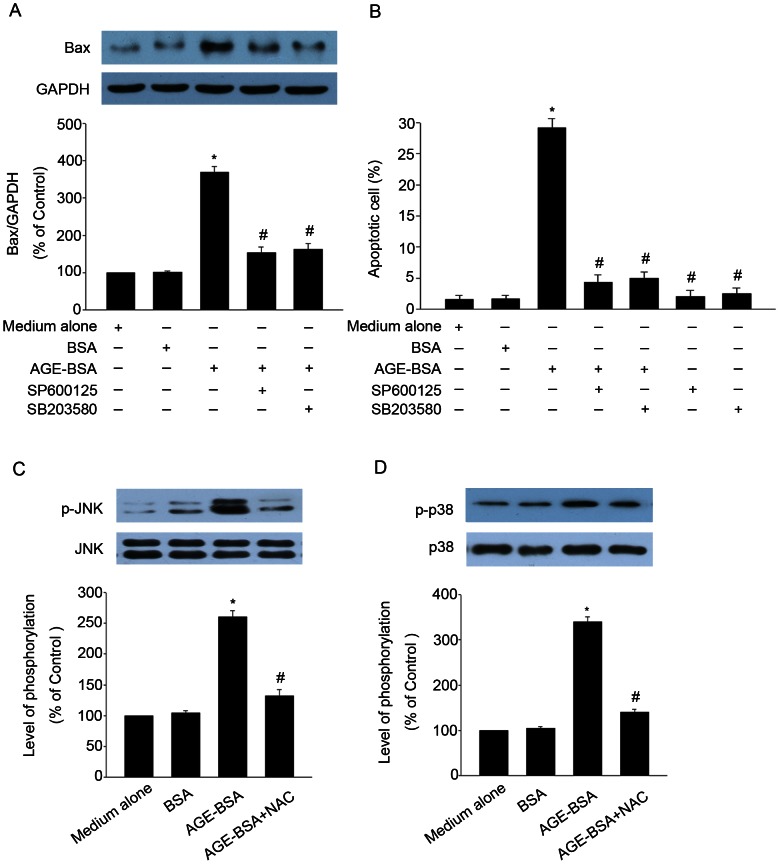
JNK and p38 MAPK mediated AGE-BSA-induced apoptosis in HUCLs. HUCLs were pretreated with JNK inhibitor (SP600125, 20 µM) or p38 MAPK inhibitor (SB203580, 20 µM) for 1 h respectively. Subsequently they were treated with AGE-BSA (200 µg/ml) for 24 h. (A) Bax protein expression was analyzed by Western blot. (B) Apoptosis was analyzed by flow cytometer. HUCLs were pretreated with ROS scavengers NAC (20 µM) for 1 h. Then the cells were stimulated with AGE-BSA (200 µg/ml) for 24 h, followed by Western blot analysis for total and phosphorylated JNK (C) and p38 MAPK (D). Data are representative of three independent experiments. *, *P*<0.05 vs. medium alone group; #, *P*<0.05 vs. AGE-BSA group.

To determine whether JNK and p38 MAPK were necessary for AGE-BSA-induced apoptosis, HUCLs were treated in the absence or presence of JNK inhibitor (SP600125) or p38 MAPK inhibitor (SB202190) for 1 h, respectively. AGE-BSA was subsequently added to the culture for 24 h. Our results demonstrated that inhibitors of JNK (SP600125) or p38 MAPK (SB203580) almost blocked Bax protein expression (Figure 8 A) and apoptosis (Figure 8 B) induced by AGE-BSA. These results showed both JNK and p38 MAPK were associated for AGE-BSA-induced apoptosis in HUCLs. To elucidate the mechanistic order of intracellular ROS production and JNK, p38 MAPK phosphorylation. HUCLs were incubated with NAC, an ROS scavenger, prior to AGE-BSA treatment. NAC almost blocked phosphorylation of JNK (Figure 8 C) and p38 MAPK (Figure 8 D) in HUCLs induced by AGE-BSA. These results suggest that intracellular ROS plays a major role as an upstream regulatory molecule in AGE-BSA-induced apoptosis in HUCLs.

## Discussion

Diabetic keratopathy has been recognized as a serious complication of diabetes. Clinically, Diabetic keratopathy may cause sight-threatening complications, such as ocular surface irregularity, microbial keratitis or even blindness. However, treatment of the disease is still lacking. The mechanism that leads to the disease is not completely understood. It was recently reported that AGEs contribute to the progression of diabetic keratopathy [14].

AGEs are a heterogeneous and complex group of products that have been involved in diabetes related complications [41]. It has been reported that enhanced AGEs accumulation contribute to diabetic ocular complications, such as diabetic retinopathy and lacrimal gland dysfunction [21], [42]. AGEs inhibitor, Aminoguanidine, has been reported to attenuate the structural alterations of the cornea in diabetic rats [11], [16]. Studies have shown that an increase in corneal epithelial cells apoptosis contributes to delayed epithelial wound healing in diabetic cornea [12], [25]. The administration of KIOM-79, AGEs inhibitor, prevented epithelial cells apoptosis in the cornea of Zucker diabetic fatty rats [13]. So, we postulate that AGE-BSA could induce corneal epithelial cells apoptosis and contributes to diabetic keratopathy. In our study, HUCLs were stimulated with AGE-BSA induced a time- and dose-dependent increase in apoptosis (Figure 1).

It is widely accepted that mitochondria plays a pivotal role in apoptotic process [43]. Apoptosis signals converge on the mitochondria via activation of pro-apoptotic members of the Bcl-2 family, such as Bax, while Bcl-2 serves as an anti-apoptotic protein [44]. Bcl-2 could neutralize the pro-apoptotic activity of Bax during apoptosis. The elevated Bax/Bcl-2 ratio could trigger apoptosis. To examine the potential mediators of AGE-BSA-induced apoptosis, HUCLs were incubated with AGE-BSA for 6, 12 and 24 h, Western blot analysis of Bax and Bcl-2 levels. Our data showed that AGE-BSA significantly increased Bax protein expression. In contrast, AGE-BSA markedly inhibited Bcl-2 protein expression (Figure 2). These results suggest that AGE-BSA-induced HUCLs apoptosis was associated with increase Bax expression and inhibite Bcl-2 expression.

RAGE is a member of the IG superfamily [45]. It is known that AGEs exert their effect through interaction with RAGE, which activates an array of various biochemical pathways [46]. AGEs-RAGE interaction has been implicated not only in diabetes but also in such settings as inflammation, hypoxia, and ischemia/reperfusion injury [47], [48]. In our study, HUCLs were stimulated with AGE-BSA induced a time- and dose-dependent increase in expression of RAGE protein (Figure 3). These results suggest that AGE-BSA may exert their pathobiological effect via RAGE on HUCLs.

Extensive evidence supports the idea that the overproduction of intracellular ROS caused by AGEs plays a important role in apoptosis [49]. AGEs induced fibroblasts apoptosis through overproduction of intracellular ROS [31]. In our study, AGE-BSA induced an acute increase of intracellular ROS generation, and the earliest significant increase in intracellular ROS production was after 3h AGE-BSA treatment in HUCLs (Figure 4 A). Moreover, AGE-BSA induced intracellular ROS production was confirmed by laser scanning confocal microscopy. AGE-BSA greatly increased the number of cells with high intensity of fluorescence compared with control (Figure 4 B). Studies have shown that there are multiple intracellular sources for the generation of intracellular ROS. NADPH oxidase, Mitochondria and xanthine oxidase have been suggested as the major sources of intracellular ROS induced by AGEs [50], [51]. However, the intracellular sources of AGE-BSA-induced intracellular ROS in corneal epithelium are not clear. We investigated the sources of intracellular ROS by assessing intracellular ROS generation under treatment of a variety of inhibitors of various enzymatic systems. Our results showed that the inhibition of NADPH oxidase with DPI or apocynin markedly suppressed intracellular ROS overproduction in AGE-BSA-treated HUCLs. In contrast, the xanthine oxidase inhibitor (allopurinol) and mitochondrial electron transport complex I inhibitor (rotenone) had no effect on intracellular ROS production (Figure 4 C), indicating that NADPH oxidase may be the most important source of intracellular ROS production induced by AGE-BSA in HUCLs. These findings agreed with the results of Yanan H [52] in which EGF-induced intracellular ROS was generated from NADPH oxidase in corneal epithelial cells. In order to find out the involvement of RAGE in AGE-BSA-induced ROS generation in HUCLs, we preincubated AGE-treated cells with anti-RAGE antibodies to block RAGE. anti-RAGE antibodies completely block the enhanced ROS generation by AGE-BSA (Figure 4 C), indicating that the essentiality of AGE-BSA-RAGE interaction in the process.

NADPH oxidase transfers electrons from NADPH to molecular oxygen and produces intracellular ROS [53]. To further investigate the mechanisms underlying the induction of intracellular ROS production by AGE-BSA, we examined the effect of AGE-BSA on the activity of NADPH oxidase. Our data showed that exposure of HUCLs to AGE-BSA induced a time-dependent increase in NADPH oxidase activity (Figure 5 A). p47phox is key cytosolic regulatory subunits of NADPH oxidase [54]. The phosphorylation of p47phox is required for the activation of AGE-BSA-induced NADPH oxidase and ROS production. Furthermore, we showed that the levels of p-p47phox were significantly increased in HUCLs 1 h after treatment with AGE-BSA (Figure 5 B), confirming the activation of NADPH oxidase in AGE-BSA-triggered HUCLs.

It was also found that HUCLs were pretreated with NADPH oxidase inhibitors (DPI or apocynin) significantly suppressed pro-apoptotic molecule Bax protein expression and apoptosis induced by AGE-BSA (Figure 6). Moreover, pretreatment of HUCLs with ROS scavenger NAC significantly inhibited apoptosis induced by AGE-BSA. These results suggest that intracellular ROS generation through NADPH oxidase was required for AGE-BSA-induced apoptosis in HUCLs. In addition, AGE-BSA induced HUCLs apoptosis could be blocked by anti-RAGE antibodies, suggesting that AGE-BSA-induced apoptosis is mainly mediated by RAGE.

JNK and p38 MAPK respond strongly to a variety of stress signals and have been implicated in mediating apoptotic responses [55]. It has been reported that AGEs induced osteoblast apoptosis via JNK and p38 MAPK [32]. AGEs stimulated fibroblasts apoptosis through JNK and p38 MAPK [31]. Based on these previous data, we hypothesized that AGE-BSA-induced HUCLs apoptosis involves JNK and p38 MAPK pathways. Our data showed that HUCLs stimulated with AGE-BSA induced activation of JNK and p38 MAPK (Figure 7). Pretreatment of HUCLs with JNK and p38 MAPK specific inhibitors (SP600125 or SB203580) almost blocked Bax protein expression (Figure 8 A) and apoptosis (Figure 8 B) induced by AGE-BSA. These results suggest that JNK and p38 MAPK were associated for AGE-BSA-induced apoptosis in HUCLs. ROS are the known mediators of intracellular signaling cascades [56]. We also found that the presence of NAC inhibited activation of JNK (Figure 8 C), and p38 MAPK (Figure 8 D) pathways. These findings suggest that intracellular ROS generation precedes the activation of JNK and p38 MAPK after AGE-BSA stimulation. Consistent with this finding, a previous study indicated that NAC almost abolished the activation of JNK and p38 MAPK in SW620 cells induced by berberine [57].

In summary, the present study demonstrated that AGE-BSA-RAGE interaction induced NADPH oxidase-dependent intracellular ROS generation, resulting in the activation of JNK and p38 MAPK pathways, which eventually leaded to apoptosis in HUCLs. Given that corneal epithelial cells apoptosis may contribute to pathologies associated with diabetic keratopathy, understanding the effects and mechanisms of AGEs on corneal epithelial cells apoptosis may provide therapeutic targets that are ultimately of clinical benefit.
